# Development of a New Marine Fish Continuous Cell Line Derived from Brain of Red Sea Bream (*Pagrosomus major*) and Its Application to Fish Virology and Heavy Metal Toxicology

**DOI:** 10.3390/ani13223524

**Published:** 2023-11-15

**Authors:** Xia Luo, Xiaozhe Fu, Min Zhang, Hongru Liang, Yinjie Niu, Qiang Lin, Baofu Ma, Lihui Liu, Ningqiu Li

**Affiliations:** 1 Pearl River Fishery Research Institute, Chinese Academy of Fishery Sciences, Key Laboratory of Fishery Drug Development, Ministry of Agriculture and Rural Affairs, Guangdong Province Key Laboratory of Aquatic Animal Immune Technology, Guangzhou 510380, China; 2 School of Marine Science and Engineering, Qingdao Agricultural University, Qingdao 266109, China

**Keywords:** *Pagrosomus major*, brain cell line, toxicology, virology

## Abstract

**Simple Summary:**

With a mass production scale and important economic and nutritive value, red sea bream (*Pagrosomus major*) is one of the most popular farmed marine teleost fish species. However, outbreaks of diseases have produced huge losses to the aquaculture industry in recent years. Fish cell lines are suitable models to study fish physiology, virology, immunology and toxicology. As the main target organ for Nervous necrosis virus infection, primary cell cultures of brain tissue were initiated for the first time from *Pagrosomus major* in this study. Our data showed that this cell line could provide wide applications for virology and toxicology for *Pagrosomus major*. RSBB cells are susceptible to Nervous necrosis virus, Singapore grouper iridovirus, Infectious spleen and kidney necrosis virus and *Siniperca chuatsi* rhabdoviruses infection. Moreover, single exposure of RSBB cells to metal Cd resulted in cytotoxicity and could induce cell apoptosis and necrosis. This study established and characterized a new marine fish brain cell line and demonstrated its suitability as a model for fish virology and toxicology.

**Abstract:**

Red sea bream (*Pagrosomus major*) is one of the most popular farmed marine teleost fish species. Fish cell lines are becoming important research tool in the aquaculture field, and they are suitable models to study fish virology, immunology and toxicology. To obtain a *Pagrosomus major* cell line for biological studies, a continuous cell line from brain of red sea bream (designated as RSBB cell line) was established and has been successfully subcultured over 100 passages. The RSBB cell line predominantly consisted of fibroblast-like cells and multiplied well in M199 medium supplemented with 10% fetal bovine serum at 28 °C. Karyotyping analysis indicated that the modal chromosome numbers of RSBB cells was 48. After transfection with pEGFP-N1, RSBB cells showed bright green fluorescence with a transfection efficiency approaching 8%. For toxicology study, it was demonstrated that metal Cd could induce cytotoxic effects of RSBB cells, accompanied with a dose-dependent MTT conversion capacity. Morphologically, cells treated with metal Cd produced rounding, shrinking and detaching and induced both cell apoptosis and necrosis. For virology study, the RSBB cells were highly susceptible to Nervous necrosis virus (NNV) and Singapore grouper iridovirus (SGIV) with steady titers (i.e., 10^8.0~8.3^ TCID_50_ mL^−1^ and 10^7.0~7.2^ TCID_50_ mL^−1^ respectively). Furthermore, an obvious cytopathic effect (CPE) could be observed in RSBB cells infected with Infectious spleen and kidney necrosis virus (ISKNV) and *Siniperca chuatsi* rhabdoviruses (SCRV). Meanwhile, all the infections were confirmed by polymerase chain reaction. The new brain cell line developed and characterized from red sea bream in this study could be used as an in vitro model for fish studies in the fields of toxicology and virology.

## 1. Introduction

Red sea bream (*Pagrosomus major*) is one of the most popular farmed marine teleost fish species, inhabiting north of the Indian Ocean to the middle of the Pacific Ocean, including China, Indonesia, Japan, Korea and the Philippines, with a mass production scale and important economic and nutritive value. Unfortunately, with the increase in marine environment pollution and intensive mariculture, outbreaks of diseases have produced huge losses to the aquaculture industry in recent years.

Fish cell lines are becoming more and more important as a research tool in the aquaculture field, and they are suitable models to study fish physiology, virology, immunology and toxicology. Since the first fish cell line RTG-2 was established by Wolf, almost 600 fish cell lines have been derived from freshwater fish or marine fish. Besides the viral diseases, marine pollution has been another constraint to the red sea beam farming industry. Among the common toxicants, toxicity of heavy metals is persistent and not easily biodegraded, and seriously endangers aquatic animals [1]. Fish have been promised as good indicators for heavy metal contamination levels in aquatic systems. Moreover, many studies interpreted that cytotoxic effects have positive correlation upon exposure to different contaminants in vitro and in vivo, indicating the importance of using fish cells in this field [2,3]. However, limited cell lines originated from brain have been reported in this field [4,5].

Viral diseases caused by red sea bream iridovirus (RSIV), nervous necrosis virus (NNV) or Singapore grouper iridovirus (SGIV) led to more than 90% mortality to *Pagrosomus major* in the fry stage [6]. A number of cell lines such as brain of striped snakehead (SSN-1), spleen of orange spotted grouper (GS) and clonal red sea bream fin-1 (CRF-1) were established to study these viruses [6,7,8]. To the best of our knowledge, only three strains of cell lines from red sea bream, including CRF-1, SBES1 and RSBF, have been established *until now* [6,9,10]. However, these cell lines derived from kidney, embryo or tail fin of seabream were not sensitive to the referred viruses.

NNV mainly infected the central nervous system of the fish, especially the retina and the brain [11,12]. Accordingly, the disease was called viral nervous necrosis (VNN) or fish viral encephalitis (FVE). Besides red sea bream, the virus could infect more than 150 species of farmed and wild fish such as turbot (*Scophthalmus maximus*), halibut (*Hippoglossus hippoglossus*), European sea bass (*Dicentrarchus labrax*) and Atlantic cod (*Gadus morhua*) [13,14,15,16]. As the main target organ for NNV infection, primary cell cultures of brain tissue were initiated for the first time from *Pagrosomus major* by the collagenase digestion method in this study. Characterization of the cell line has been identified. In addition, we tested its application to aquaculture virology by evaluating the viral susceptibility to NNV, SGIV, Infectious spleen and kidney necrosis virus (ISKNV) and *Siniperca chuatsi* rhabdoviruses (SCRV). Furthermore, we evaluated its potential use in metal toxicology by exposing the cells to Cd. Our data showed that this cell line could provide wide applications for virology and toxicology for *Pagrosomus major.*

## 2. Materials and Methods

### 2.1. Establishment and Subculture of the RSBB Cell Line

Red sea bream with a total length of 10 cm were obtained from a local marine fish farm in Yangjiang City, Guangdong Province. To make sure the fish were without special pathogens, pathogens including RSIV and NNV were detected before the experiment. The method of developing the RSBB cell line referred to that described for the CPB cell line with some modifications [17]. In accordance with the animal rights law, the fish were anesthetized firstly and then disinfected with 75% *v*/*v* alcohol. The fish were dissected in sterile conditions and brain was obtained aseptically and minced to *c.* 1.0 mm^3^ tissue blocks. After being washed to remove blood cells, the blocks were then incubated in Leibovitz’s L-15 medium supplemented with 10% foetal bovine serum (FBS), 0.1% collagenase (Type) and antibiotics (200 IU mL^−1^ penicillin, 200 μg mL^−1^ streptomycin and 0.5 μg mL^−1^ amphotericin B) at 28 °C for 4–6 h until dispersed into single cells. Subsequently, the suspension was centrifuged at 200× *g* for 10 min. The pellet was re-suspended in Leibovitz’s L-15 medium containing 20% FBS, 100 IU mL^−1^ penicillin, 100 μg mL^−1^ streptomycin and 0.25 μg mL^−1^ amphotericin B, 10 ng mL^−1^ epidermal and basic fibroblast growth factor, and inoculated into 25 cm^2^ tissue culture flasks maintaining at 28 °C. Half of the medium was replaced every 3–4 days.

When reached monolayer, the primary cells were trypsinized with commercial 0.25% trypsin-EDTA solution (Gibco, Carlsbad, CA, USA) and subcultured at a ratio of 1:2. Detached cells were re-suspended in complete L-15 medium with 20% FBS, 100 IU mL^−1^ penicillin, 100 μg mL^−1^ streptomycin and 0.25 μg mL^−1^ amphotericin B. After 5 passages, the growth medium was changed to L-15 with 10% FBS.

### 2.2. Characterization of the RSBB Cell Line

#### 2.2.1. Cryopreservation Efficiency

For cryopreservation, when grew at the logarithmic phase, RSBB cells at different passages from 5 to 65 (every five passages) were stored in liquid nitrogen. A detailed procedure was carried out as described previously [17]. Finally, the cell cultures were stored in liquid nitrogen for long-term storage.

For revival, a cryovial was thawed quickly in a water bath at 30 °C. Then, the tube was centrifuged at 500× *g* for 5 min at room temperature and re-suspended in M199 medium supplemented with 10% FBS and seeded into a 25 cm^2^ tissue culture flask at 28 °C.

#### 2.2.2. Immunophenotyping Characterization

Mouse anti-fibronectin antibodies (Sigma, Rahway, NJ, USA) and mouse anti-cytokeratin antibodies (Sigma, USA) were used to determine the immunophenotyping of RSBB cells at the 25th passage. The detailed procedure was according to the previous literature [18]. Briefly, after cultured overnight in 6-well plates, RSBB cells were fixed with methanol for 20 min. Then the cells were washed with PBS twice. Afterwards, the above two primary antibodies and second antibody (rabbit anti-mouse IgG, Sigma, USA) were incubated successively. And the cells were examined using an inverted fluorescence microscope.

#### 2.2.3. Chromosome Analysis

When grown for 24 h, RSBB cells from passages 15 and 60 were treated with 0.2 μg mL^−1^ colcemid (Sigma, USA) for 4 h at 28 °C to arrest cells at the metaphase. After harvested by centrifugation at 200× *g* for 5 min, cells were treated with hypotonic solution of 0.075 M KCl for 25 min, and then fixed in 3:1 of methanol: acetic acid for 15 min at room temperature. According to the conventional drop-splash technique by Freshney (1994) [19], slides were prepared and then stained with 5% fresh Giemsa solution for 20 min (Sigma, USA). Chromosomes of at least 100 metaphase cells from passages 15 and 60 were counted by phase-contrast microscope (Leica, Weztlar, Germany).

#### 2.2.4. Cell Growth Characteristics

To determine the optimum cell culture medium and FBS concentration, RSBB cells at an initial density of 1 × 10^5^ cells mL^−1^ were seeded into 12 well plates with 3 different mediums including L-15, Dulbecco’s modified Eagle’s medium (DMEM) and M199 containing 10% FBS at 28 °C. At an interval of 24 h, cells from triplicate wells were trypsinized and counted with an automated cell counter (Count star, Shanghai, China) for 6 days. Similar procedures were carried out to optimize the concentration of FBS (6, 8 and 10%) on cell growth.

#### 2.2.5. Cell Transfection

RSBB cells at passage 60 were used for transfection analysis. Firstly, RSBB cells were propagated in a 24 well plate at a density of 1 × 10^5^ cells well^−1^. Subconfluent monolayers were transfected with 2 μg of pEGFP-N1 eukaryotic expression vector (Invitrogen, Carlsbad, CA, USA) using lipofectamine 2000 (Invitrogen, USA). Twenty-four hours later, green fluorescence was observed under a Leica fluorescence microscope.

### 2.3. Virology

#### 2.3.1. Virus Sensitivity

NNV, SGIV, ISKNV and SCRV isolated from diseased *Pagrosomus major*, *Epinephelus coioides* and *Siniperca chuasi* in the Pearl River Delta in China, purified and stored in our laboratory, were used to test the viral sensitivity of the RSBB cell line here. After overnight culturing, confluent monolayers of RSBB cells were infected with the viruses at a multiplicity of infection (MOI) of 0.1 for NNV, 1 for ISKNV and 0.01 for SGIV, SCRV, respectively. Briefly, after 1~2 h absorption, unattached viruses were removed and the M199 medium with 2% FBS was supplemented to the culture plates. CPE caused by the viruses were observed daily using an inverted light microscope. When the CPE reached about 80%, samples were harvested after the freeze-thaw process twice at −80 °C. For viral titration, the 50% tissue culture infective dose (TCID_50_) method was used in a 96-well culture plate according to the method of Reed and Muench (1938) [20].

#### 2.3.2. PCR Detection for the Viruses

Virus infection in RSBB cells was confirmed by PCR analysis using gene-specific primers based on the capsid protein gene for NNV, the major capsid protein (MCP) gene for SGIV and ISKNV and the G protein gene for SCRV, which were registered in GenBank (Table 1). The PCR products were analyzed in 1% agarose gels containing 0.5 mg mL^−1^ ethidium bromide and sequenced by Shanghai Sang-gong Biological Engineering Technology & Services Co., Ltd. (Shanghai, China) (www.sangon.com (accessed on 10 May 2021)).

### 2.4. Metal Cytotoxicity to RSBB Cells

#### 2.4.1. Cadmium Chloride (CdCl_2_) Exposure and Viability Test of RSBB Cells

For toxicity test, RSBB cells were cultured overnight in 96-well plates with an initial density of 2 × 10^5^ cells well^−1^, following washes in fresh medium. Then, the cells were inoculated with 200 μL serial dilutions of CdCl_2_ (180 μLM199 with 5% FBS + 20 μL CdCl_2_) to achieve final concentrations of 0.001, 0.005, 0.01, 0.05, 0.1 and 0.5 mmol/L. For negative controls, cells were seeded in 200 μL M199 with 2% FBS. Twenty-four hours after the exposure at 28 °C, the cell viability was measured by the MTT colorimetric method according to the manufacturer’s methods (Sigma). The experiment was performed in triplicate and repeated three times. The data were expressed as means ± standard deviation (*n* = 3). *t* test was used to determine significant differences. All the statistical analyses were done with the SPSS ver. 20 statistical software package (IBM Corp., Armonk, NY, USA).

#### 2.4.2. Cell Apoptosis or Necrosis Induced by CdCl_2_

To investigate whether cadmium can induce apoptosis or necrosis in RSBB cells, the Annexin V-FITC/PI staining kit (TransGen Biotech, Beijing, China) was used as described previously [4,21]. Briefly, when grown to a monolayer, the cells were exposed to a final dosage of 0.1 mmol/L Cd based on our preliminary experiments for 24 h at 28 °C. Then, RSBB cells were incubated with the detection kit solution in the dark for 15 min at room temperature. Finally, the cells were observed using an inverted fluorescence microscope. The experiment was performed twice with duplicated samples.

## 3. Results

### 3.1. Primary Culture and Subculture of RSBB Cells

**Figure 1:**
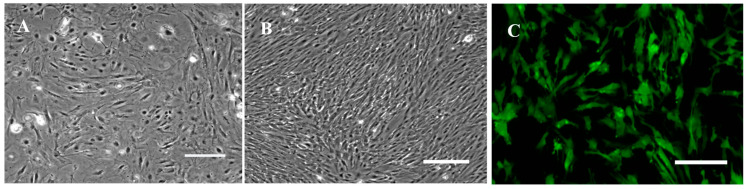
Morphology and immunophenotype characterization of RSBB cells. (**A**) Morphology of RSBB cells at the 1st passage. (**B**) Morphology of RSBB cells at the 24th passage. (**C**) RSBB cells at the 25th passage produced fibronectin marker. Scale bar = 200 μm.

With the collagenase digestion method, primary cells were derived from brain of *Pagrosomus major*. RSBB cells grew in a confluent monolayer in about 20 days. Cells were cultured in L-15 medium containing 20% FBS at 1:2 at 5 day intervals for the first 10 passages. Then, cells grew rapidly and FBS was reduced to 10%. At this period, cells were subcultured at 1:3 every 4 days. When they reached the 20th passage, the cells were split at 1:3 at 3-day intervals. So far, the cell line had been subcultured > 80 times and was designated as the red sea bream brain cell line (RSBB). Morphologically, original cell cultures were composed of epithelial and fibroblastic cells (Figure 1A), while fibroblast-like cells dominated after 20 subcultures (Figure 1B). To confirm the immunophenotyping of RSBB cells, strong fluorescence was detected in RSBB cells at passage 25 when incubated with mouse anti-fibronectin antibodies (Figure 1C), while no signal appeared in cells incubated with mouse anti-cytokeratin antibodies or in control cells. The results also demonstrate that RSBB cells are fibroblast-like cells.

### 3.2. Cell Thawing Efficiency of Cryopreserved RSBB Cells

RSBB cells were cryopreserved in liquid nitrogen every five passages (from 5th to 65th passage) and thawed after 1 year of storage. The cells showed viability of more than 80% and grew in monolayer within 3–4 days in M199 with 10% FBS. There was no alteration for RSBB cells in aspects of morphology and growth rate after freezing and thawing.

### 3.3. Effect of Culture Medium and FBS on Growth of RSBB Cells

**Figure 2:**
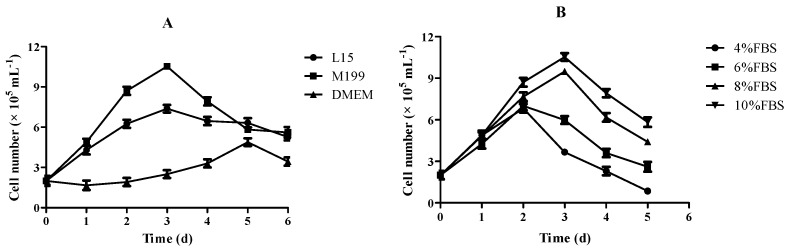
Growth kinetics of RSBB cell line. (**A**) Effects of different growth media supplemented with 10% FBS at 28 °C on cell growth. (**B**) Effects of different FBS concentrations on cell growth in M199 at 28 °C on cell growth.

To optimize the culture conditions, growth kinetics of the RSBB cell line at the 50th passage were studied with different culture mediums and FBS concentrations. Cells exhibited different growth rates in different culture media at 28 °C (Figure 2A). Results showed that M199 was the most suitable culture medium and RSBB cells could also grow in L-15, while there was almost no growth until the fifth day when cells grew in DMEM. The growth rate of RSBB cells increased along with FBS proportion from 4 to 10% at 28 °C. Poor growth of the cells was observed at 4% and 6% FBS, while relatively good growth was observed at 8% FBS. When FBS was enhanced to 10%, a maximum growth occurred (Figure 2B).

### 3.4. Chromosome Analysis

**Figure 3:**
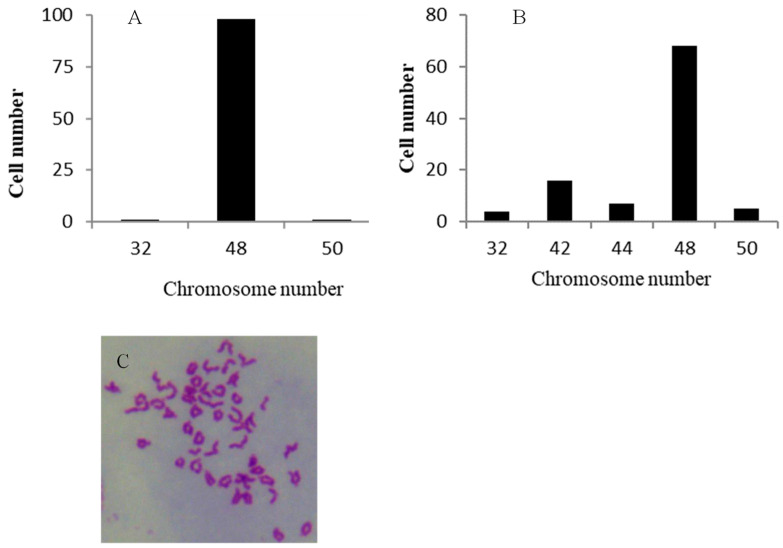
Chromosome number distribution of RSBB cell line. (**A**) passage 15. (**B**) passage 60. (**C**). Metaphase of RSBB cell line. In total, 100 metaphases were counted.

One hundred metaphase plates from RSBB cells at the 15th and 60th passage were used for chromosome counts. At the 15th passage, 98% of the RSBB cells contained 48 chromosomes (Figure 3A). While at the 60th passage, the chromosome number ranged from 32 to 50, with a modal number of 48 (Figure 3B). Our data showed almost all of the chromosomes were telocentric (Figure 3C).

### 3.5. Transfection Efficiency

**Figure 4:**
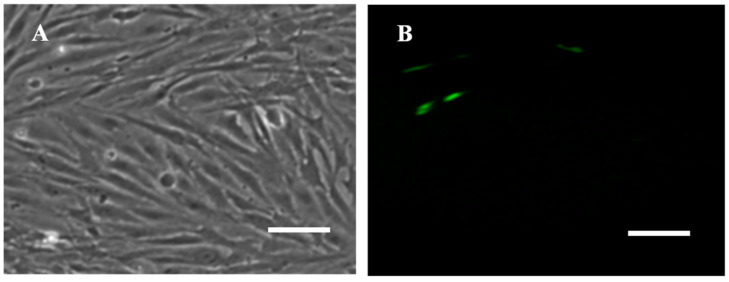
(**A**) Fluorescent and (**B**) optical microscope photographs of RSBB cells transfected with pEGFP-N1plasmid at passage 60. Scale = 200 μm.

The RSBB cells were successfully transfected with pEGFP-N1 expression plasmid. Clear and strong green fluorescence signals could be detected at 24 h post transfection with a transfection efficiency of about 8% (Figure 4).

### 3.6. Virus Susceptibility

**Figure 5:**
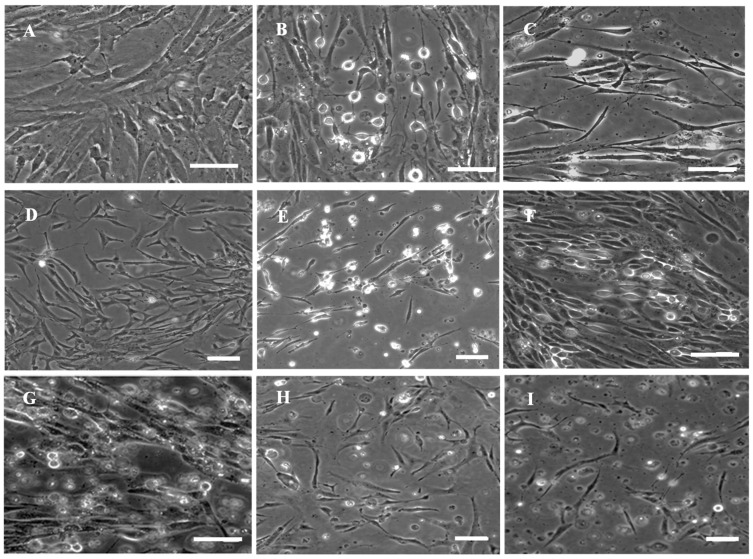
Cytopathic effects of the RSBB cells after infection with NNV, SGIV, ISKNV and SCRV by invert light microscopy. (**A**) mock-infected RSBB cell, (**B**) 48 h pi with NNV, (**C**) 72 h pi with NNV, (**D**) 24 h pi with SGIV, (**E**) 48 h pi with SGIV, (**F**) 48 h pi with ISKNV, (**G**) 96 h pi with ISKNV, (**H**) 42 h pi with SCRV, (**I**) 48 h pi with SCRV. Scale bar is 50 μm in (**D**,**E**,**H**,**I**) and 20 μm in (**A**–**C**,**F**,**G**). Scale bar = 200 μm.

The RSBB cell line was tested for its susceptibility to NNV, SGIV, ISKNV and SCRV. After infection with the viruses, RSBB cell cultures showed CPE in all cases within 7 days, whereas the control cell cultures were normal, demonstrating great susceptibility (Figure 5A). The morphological changes of the RSBB cells at different infection stages are shown in Figure 5B–I. Following inoculation with NNV for 48 h, cytopathic effects such as cell shrinkage and rounding were observed, and then many of the RSBB cells detached from the flask at 96 h post infection (h.p.i.) (Figure 5C). When RSBB cells were inoculated with SGIV, advanced CPE such as cell lysis was observed in RSBB cell cultures at 24 h.p.i (Figure 5D). As CPE development progressed, some cells became round and more and more cells detached from the culture flask (Figure 5E). Cell enlargement and clustering were the typical CPE after ISKNV infection. Firstly, cell shrinkage and rounding appeared at 48 h.p.i (Figure 5F). Then infected cells became enlarged and clustered, and lots of cells detached from the flask at 96 h.p.i (Figure 5G). When RSBB cells were inoculated with SCRV, more and more cells became round within 42~48 h, accompanied with drawbench and detachment (Figure 5H–I). At last, a large number of the infected cells detached with cultures being completely destroyed at 4 days post infection (d.p.i) (Figure 5I).

In addition, the viral titers of NNV and SGIV were tested at the 50th, 55th and 60th passages of the RSBB cell line and the titers reached 10^8.0~8.3^ and 10^7.0~7.2^ TCID_50_ mL^−1^ within 7 days, respectively. The results demonstrated that the sensitivity and replication efficiency of the viruses were relatively stable and superior, which proved that the RSBB cell line was hypersensitive to the viruses.

### 3.7. PCR Detection for Virus Specificity

**Figure 6:**
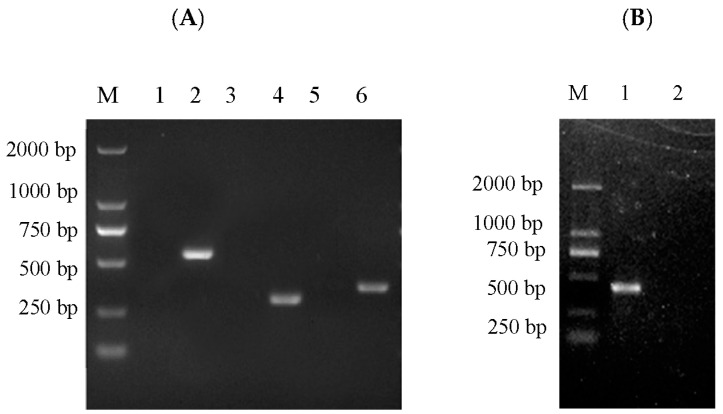
PCR detection for virus specificity (**A**): M. Marker 2000, 1. ISKNV (negative control); 2. supernatant of RSBB cells infected with ISKNV; 3. SCRV (negative control); 4. supernatant of RSBB cells infected with SCRV; 5. SGIV (negative control); 6. supernatant of RSBB cells infected with SGIV; (**B**): M. Marker 2000, 1. supernatant of RSBB cells infected with NNV; 2. NNV (negative control).

Virus infection was confirmed by PCR amplification. Viral genome of ISKNV, SCRV, SGIV and NNV was extracted separately from liquid supernatant of infected RSBB cell lines. Then PCR detection was carried out; the target bands of 562 bp, 317 bp, 372 bp and 420 bp were obtained for the above samples, respectively (Figure 6). Sequence analysis showed high homologies (more than 99.9%) to the corresponding sequences registered in GenBank, demonstrating the infectivity of these viruses to RSBB cells.

### 3.8. Cell Vitality Affected by Cd

**Figure 7:**
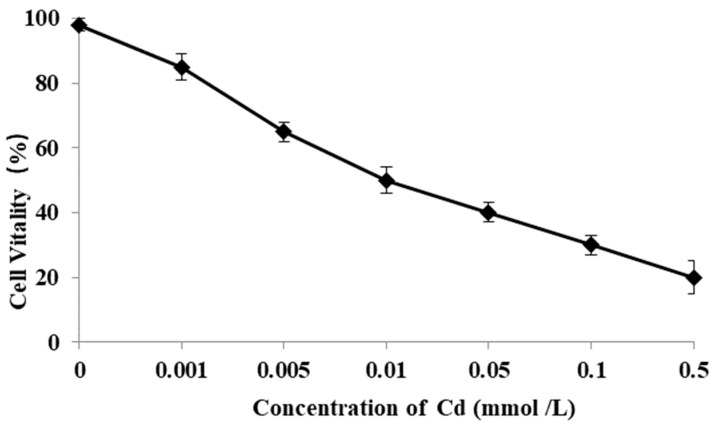
Cell vitality affected by Cd.

Cell vitality of RSBB was evaluated after exposure to metal Cd with different concentrations for 24 h. Results showed that cell vitality reduced when the dose of Cd increased, accompanied with a dose-dependent manner (Figure 7). Moreover, obvious differences appeared when the concentration of Cd exceeded 0.001 mol/L (*p* < 0.01).

### 3.9. Cell Apoptosis and Necrosis Induced by Cd

**Figure 8:**
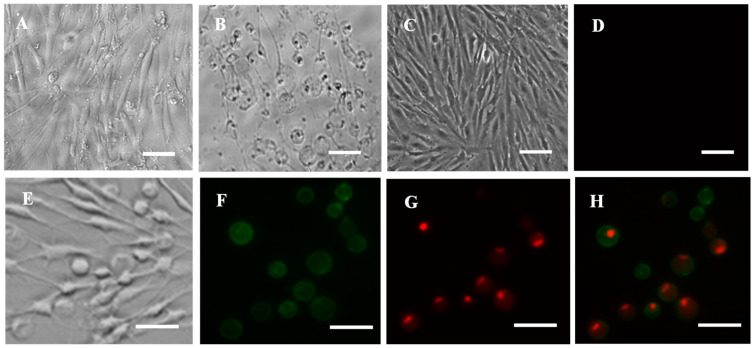
Cytotoxic effect of Cd to RSBB cells.(**A**) Cytotoxic effect of Cd to RSBB cells at 24 hpi; (**B**) Cytotoxic effect of Cd to RSBB cells at 48 hpi; (**C**) Mock RSBB cells; (**D**) Cellular apoptosis detection in mock RSBB cells using annexin V-FITC/PI staining, no green or red signal appeared; (**E**) Optical microscope photograph of Cd treated RSBB cells; (**F**) Cellular apoptosis detection in Cd-treated RSBB cells using annexin V-FITC/PI staining, red fluorescence represents necrosis; (**G**) Green fluorescence represents early apoptosis; (**H**) Merged. Scale = 200 μm.

In addition, cells treated with metal Cd produced rounding, shrinking, detaching and finally monolayer destruction (Figure 8A,B), while it was normal in control cells (Figure 8C). Annexin V-FITC/propidium iodide (PI) double staining was used to determine the cellular apoptosis and necrosis of RSBB cells when treated with Cd. Results showed that both green and red signals were observed in Cd-treated RSBB cells after 24 h post infection (pi) (Figure 8E–H), while no green or red signal appeared in control cells, suggesting both apoptosis and necrosis were induced at an early stage (Figure 8D).

## 4. Discussion

Although red sea bream is one of the most important fish species for the Mediterranean aquaculture industry, the availability of cell lines is very limited, and there is no description in the literature about brain-derived cell lines in this species. In this study, a new marine fish cell line (RSBB) from brain of red sea bream has been successfully established and characterized, which will provide a useful tool for virology and toxicology studies.

The brain tissues of teleost fish are favorable for developing cell lines since they have numerous proliferating cells. High rates of neurogenesis and regeneration appeared after injury [22]. Thus, more and more brain-derived cell lines such as RGB [23], OnlB [18], CPB [17], EMB [24], CAMB [25] and SaB-1 [26] have been reported recently. To the best of our knowledge, this is the first report about the development of a brain cell line from red sea bream.

In the primary culture, RSBB cells showed mixed morphologies including epithelial-like and fibroblast-like cells. After 20 subcultures, fibroblast-like cells ultimately predominated and maintained for more than 50 passages so far, which was consistent with RSBF and CRF-1 cell lines developed from the same species and some other brain cell lines [6,9,17]. Many studies have revealed that L-15 is preferable for the development of cell cultures from fishes [27]. However, our results showed that RSBB cells grew well in M199 supplemented with 10% FBS. The chromosomal number is an important feature to characterize cell lines. Kidney and tail fin cell lines of red sea bream have a diploid chromosomal number of 2*n* = 48 [6,9,28]. In this study, 98% of the RSBB cells contained 48 chromosomes at passage 15, when at passage 60, the chromosome number ranged from 32 to 50 with a modal number of 48, demonstrating a consistent chromosome number to previous reports.

In vitro toxicology fish cell lines have been used for many toxicological studies [4,26]. Metal exposure produced alterations in the proliferation of neural cells and induced cell apoptosis and necrosis [29,30]. According to the previous literature, Cd was evaluated as one of the most toxic metals to several fish cell lines such as RTG-2, Sub-G1, DLB-1 and SAF-1 [4,29,31]. Thereby, Cd was used to test the cytotoxic effect to RSBB cells in this study. Cell viability of RSBB was affected as early as 24 h post exposure to metal Cd, accompanied with a dose-dependent manner. Meanwhile, our data showed that RSBB cells exposed to Cd died by apoptosis and necrosis as demonstrated by annexin V/PI staining and DAPI staining, which is consistent with the previous literature. In Krumschnabel’s study, apoptosis and necroptosis were induced in rainbow trout cell lines after exposure to cadmium [29]. Morcillo [30] reported Cd promoted both apoptosis and necrosis cell death to the head-kidney leucocytes (HKLs) of European sea bass (Dicentrarchus labrax). However, only apoptosis without necrosis cell death was detected in DLB-1 cell lines after exposure to Cd, Me, Hg, Pb and As [4]. As for the mechanism, caspase activation, DNA fragmentation, ATP depletion and plasma membrane permeabilization have been reported as typical characterizations for cell apoptosis and necrosis, which were dependent on the cell type and concentration of heavy metals. Subsequently, further studies are needed to ascertain the cytotoxicity mechanisms induced by metals in RSBB cells.

Nowadays, viral infections are responsible for high rates of mortality in both marine and freshwater fish. For this reason, the susceptibility of RSBB cells to four highly infectious and noticeable viruses, which cause economic losses in modern aquaculture and affect a wide range of host species, was analyzed. The four viruses were NNV, which affects more than 150 marine fish species [13,14,15,16,32,33], SGIV, which mainly affects Grouper [7], ISKNV and SCRV, which mainly affect mandarin fish and largemouth bass [34,35]. For the diagnosis and infection mechanism studies of NNV, some cell lines, such as GF-1, SF, SSN-1, GS, have been established [7,8,11,12,36]. Among these, the GS cell line reported by Qin [7] was sensitive to both NNV and SGIV, with a high titer of 10^7.2^TCID_50_ mL^−1^ and 10^7.5^TCID_50_ mL^−1^, respectively [7]. Here in this study, viral titers in RSBB cells reached 10^8.0~8.3^ and 10^7.0~7.2^ TCID_50_ mL^−1^, demonstrated high sensitivity and replication efficiency for the viruses and would be useful for the propagation of these viruses. Furthermore, the RSBB cell line was also sensitive to the other three freshwater viruses, with obvious CPE of swelling, drawbench and detachment. These results indicate that the cell line is useful for the isolation and detection of viruses in fish from this region.

## 5. Conclusions

In this study, we have established a continuous brain cell line (RSBB) from red sea bream. RSBB cells grew well in M199 containing 10% FBS. It is susceptible to NNV, SGIV, ISKNV and SCRV infection. Moreover, single exposure of RSBB cells to metal Cd resulted in cytotoxicity and could induce cell apoptosis and necrosis. This study established and characterized a new marine fish brain cell line and demonstrated its suitability as a model for fish virology and toxicology.

## Figures and Tables

**Table 1:** Primers for PCR amplification.

Virus	Primer Sequences	Accession Number	Fragment Length/bp
NNV	R3:5′-CGAGTCAACACGGGTGGAGA-3′	MN496376.1	420
	F2:5′-CGTGTCAGTCATGTGTCGCT-3′		
SGIV	F:5′-CGGGCAAGAGTTTTCGGTC-3′	MK107821.1	372
	R:5′-AACGGCAACGGGAGCACTA-3′		
ISKNV	F:5′-CGTGAGACCGTGCGTAGT-3′	AF371960	562
	R:5′-AGGGTGACGGTCGATATG-3′		
SCRV	F:5′-CTGAATCTCCAAGAATGGAAAACC-3′	DQ399789.1	317
	R:5′-AATTTTGGCCAACTCGATTC-3′		

## Data Availability

Data supporting this study are available in the article.
